# CD44 Knockout Alters the Cellular Response and Improves Locomotor Outcome After Spinal Cord Injury

**DOI:** 10.1080/17590914.2026.2673257

**Published:** 2026-08-27

**Authors:** Dana Creasman, Francisca Benavente, Rebecca Nishi, Aileen Anderson

**Affiliations:** a Sue and Bill Gross Stem Cell Research Center, University of California Irvine, Irvine, CA, USA; b Department of Anatomy and Neurobiology, University of California, Irvine, CA, USA; c Center of Regenerative Medicine, Facultad de Medicina, Universidad del Desarrollo, Santiago, Chile; d GMP Facility, University of California Irvine, Irvine, CA, USA; e Institute for Memory Impairments and Neurological Disorders, University of California Irvine, Irvine, CA, USA

**Keywords:** Spinal cord injury, Neural stem cells, Complement, C1q, Cd44, Neuroimmune

## Abstract

Spinal cord injury (SCI) is a devastating condition that results in long-term functional impairments due to loss of tissue and limited regeneration. Investigating the factors that regulate the post-SCI response is critical to understanding the pathophysiology of this condition and developing treatments. One molecule of interest in the post-SCI response is CD44. CD44 is cell-surface protein with a well-established role in regulating cellular functions including cell migration and proliferation. CD44 is expressed in many cells that play a role in the post-SCI microenvironment but the effect of global CD44 KO on SCI outcomes has not previously been tested. Here, we investigate that role in a mouse unilateral cervical contusion SCI model. We predicted that CD44 KO would exert a predominant effect on glial progenitor/cell recruitment, inhibiting astroglial scar formation and thereby increasing lesion expansion and exacerbating locomotor deficits. In contrast, we found that CD44 KO mice exhibited increased numbers of astrocytes at the lesion epicenter, developed a more compact astroglial scar by 28 days after injury, showed improved locomotor function, and exhibited decreased recruitment of acutely activated immune cells vs WT mice. Together, these findings highlight the role of CD44 in diverse cell populations after SCI as a critical point of further investigation to understand the post-SCI response.

## Summary Statement

In a spinal cord injury model, CD44 knockout mice exhibited increased numbers of astrocytes at the lesion epicenter, developed a more compact astroglial scar by 28 days after injury, showed improved locomotor function, and exhibited decreased recruitment of acutely activated immune cells vs wildtype mice.

## Introduction

Spinal cord injury (SCI) is a devastating condition that results in long-term functional impairments due to loss of tissue and limited regeneration (Norenberg et al., 2004). SCI creates a sharp decrease in length and quality of life for those affected, along with a sizeable healthcare cost (University of Alabama at Birmingham, 2021). This is compounded by the lack of robust therapeutics for SCI. A critical step towards developing better SCI treatments is developing a more complete understanding of the complex biological response to SCI. In broad terms, SCI consists of an initial primary insult to the cord, followed by a secondary damage response over the following days/weeks (Ahuja et al., 2017; Anwar et al., 2016; Norenberg et al., 2004). This secondary injury is the net product of a wide variety of different cell types, including resident and infiltrating immune cells and astrocytes, and is modulated by pro-regenerative responses, including the activation of endogenous stem/progenitor cells (Anwar et al., 2016; Fleming et al., 2006; O’Shea et al., 2017; Whetstone et al., 2003; Zai & Wrathall, 2005). An early event in this process is the breakdown of the blood-spinal cord-barrier (BSB), which results in an influx of humoral components into the previously “immune privileged” spinal cord (Bartanusz et al., 2011; Figley et al., 2014; Matsushita et al., 2015; Whetstone et al., 2003). This leads to a rapidly altered immune microenvironment, including a prolonged, multiphasic infiltration/activation of immune cells such as macrophages/microglia and neutrophils, and an influx of complement proteins (Beck et al., 2010; Fleming et al., 2006; Galvan et al., 2008; Hooshmand et al., 2009, 2017). In conjunction with this, native astrocytes, stem/progenitor cells, and microglia are activated to form a glial scar barrier between the lesion site and spared spinal cord tissue, and to restore myelination by incorporation of new oligodendrocytes (Anderson et al., 2016; Barnabé-Heider et al., 2010; Bellver-Landete et al., 2019; Johansson et al., 1999; Meletis et al., 2008; O’Shea et al., 2017; Powers et al., 2013; Sabelström et al., 2013, 2014; Stenudd et al., 2015; Wanner et al., 2013; Zhou et al., 2023).

A potential shared common actor across several of these processes is the cell-surface protein CD44, a receptor with an established role regulating cell behavior in astrocytes and astrocyte progenitors, oligodendrocyte progenitors, and neural stem cells (NSC) (Cichy & Puré, 2003; Dzwonek & Wilczynski, 2015; Marhaba et al., 2008; Morath et al., 2016; Ponta et al., 2003; Senbanjo & Chellaiah, 2017; Su et al., 2017; Yan et al., 2015). CD44 is alternatively spliced to generate multiple variants, with the standard form of CD44 (CD44s) containing exons 1–5 and 16–20 (other variants will include one or more of exons 6–15) (Ponta et al., 2003). CD44 has a diverse set of ligands, including hyaluronan (HA), osteopontin, and matrix metalloproteases. Consistent with this variety of forms and ligands, CD44 has a multitude of functions. It is involved in both cell-cell and cell-matrix interactions, and cell migration, proliferation, and fate (Dzwonek & Wilczynski, 2015; Ponta et al., 2003; Weber et al., 1996). CD44 has been previously shown to modulate the behavior of multiple cell types in the CNS, especially astrocytes (Dzwonek & Wilczynski, 2015), and linked to astroglial differentiation, reactive astrogliosis, and regulation of the astroglial cytoskeleton (Bourguignon et al., 2007; Shaltouki et al., 2013; Zamanian et al., 2012). CD44 also has established functions in NSC. HA- CD44 is expressed by spinal-cord derived NSC (Cusimano et al., 2018), and CD44 signaling negatively regulates proliferation in brain NSC both in vitro and in vivo (Su et al., 2017). Consistent with these data, CD44 has been identified as a receptor for the complement protein C1q, and shown that C1q interaction with CD44 induces NSC migration both in vitro and after NSC transplantation [Benavente]. Critically, CD44 KO in transplanted hNSC blocked these cells from migrating to the SCI lesion epicenter and differentiating into astrocytes. Additionally, CD44 expression in not only astrocytes, but microglia (especially activated microglia) as well has been shown to be relevant in neurodegenerative conditions like ALS and stroke (Matsumoto et al., 2012; Sawada et al., 2020). Together these data suggest that CD44 could play a multi-faceted role in the secondary injury response in the damaged spinal cord. Critically, a recent study has directly shown that inhibiting CD44 function impacts scar formation post-SCI, directly providing evidence for CD44’s important role in this context (Guo et al., 2024).

In the present study, we further tested the role of CD44 in the spinal cord injury response after cervical contusion in a murine model of global CD44 KO, investigating histological factors including cell proliferation, astroglial scar formation, lesion volume, and the immune cell response, as well as recovery of locomotor function. We predicted that CD44 KO would exert a predominant effect on glial progenitor/cell recruitment, inhibiting astroglial scar formation and thereby increasing lesion expansion and exacerbating locomotor deficits. In contrast, we found that CD44 KO mice exhibited increased numbers of astrocytes at the lesion epicenter, developed a more compact astroglial scar by 28 days after injury, showed improved locomotor function, and exhibited decreased recruitment of acutely activated immune cells vs WT mice. Together, these results indicate a wide-reaching role for CD44 in the regulating the post-SCI response and highlight CD44 not only as a target for further study in elucidating the basic biology of SCI, but also as a potential therapeutic target to improve histological and functional outcome.

## Materials and Methods

### Animal Models

All experiments were carried out in accordance with the Institutional Animal Care and Use Committee at [details omitted for double-anonymized peer review], and were consistent with Federal guidelines. CD44 KO mice (RRID:IMSR_JAX:005085) and wildtype control mice (RRID:IMSR_JAX:000664) were crossed to create heterozygous animals, and heterozygous animals crossed to produce KO and WT littermates for experimental use. A total of 46 animals were used between the two genotypes and three sacrifice timepoints. See Table 1 for a list of and rationale for subject exclusions for this study.

**Table 1.** Animal exclusions.

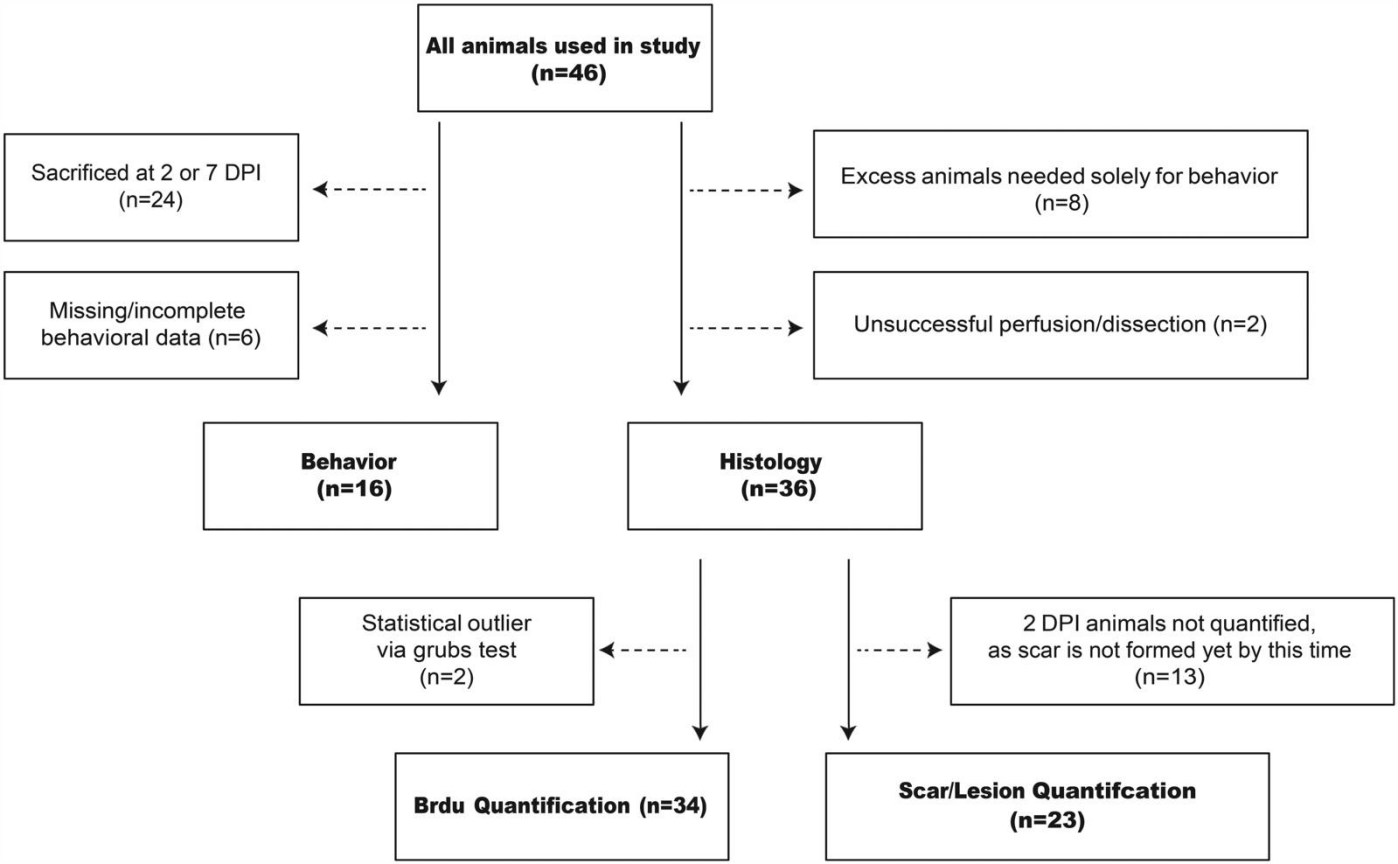

Solid arrows indicates animals included in given analysis, dashed arrows indicated animals excluded from given analysis.

### Surgery

15 week old mice were anesthetized for surgery using 2.5% isoflurane; all mice received a laminectomy at the C5 vertebral level. Right-sided, unilateral contusion injuries were induced using the Infinite Horizon Impactor (Precision Systems and Instrumentation), using a force of 30kD and dwell time of 5s, as described previously (Nishi et al., 2020). A cervical model of SCI was chosen due to the majority of human cases of SCI occurring at the cervical level (Alizadeh et al., 2019).

### BrdU Injections

BrdU (Sigma) was injected into animals intraperitoneally at 12, 24, and 36 hours post-SCI, at a concentration of 10 mg/kg of animal body mass.

### Histology and Stereological Analysis

At 2DPI, 7DPI or 28DPI mice were terminally anesthetized and transcardially perfused with 30 ml PBS, followed by 75 ml 4% PFA. Spinal cord regions corresponding to dorsal roots at C1-T2 were dissected and cryoprotected overnight in 4% PFA/20% sucrose, flash frozen at −55 °C in isopentane, and stored at −80 °C until tissue processing. Spinal sections were embedded in Neg50 Frozen Section Medium (Thermo) and 30 µm thick transverse-coronal sections cut on a Cryostat using a CryoJane tape transfer system (Leica), as previously described (Sontag et al., 2014). Frozen sections were collected on slides and stored at −20 °C until processed for immunohistochemistry. All sectioning and immunohistochemistry procedures were conducted as previously described (Anderson et al., 2017; Benavente et al., 2020; Nishi et al., 2020; Sontag et al., 2014). Dilutions used in immunohistochemistry are listed in Table 2. Unbiased histological quantification was conducted using stereology by investigators blinded to experimental groups. A random set of animals (*n* = 5–6/group) were selected for cell counts. The optical fractionator probe was used for estimation of the number of BrdU+, GFAP+, PU.1+, and Sox9 positive cells. in StereoInvestigator (Microbrightfield, give software version). Grid size measurements were determined based on preliminary experimentation to establish a low coefficient of error (CE < 0.1). The central canal was treated as a separate region from the parenchyma for the sake of counts. Cells had to be contiguous with the ring structure of the canal to be considered part of the canal. Cell migration was reported as the total estimated number of cells per section, in a distribution graph where the injury epicenter is defined as the area of the cord with the largest Fibronectin positive area. A Cavalieri probe was used to estimate total volumes of fibronectin + lesion volume, in 1 of 6 intervals from coronal spinal cord sections 360 µm apart, at 10× magnification.

**Table 2.** List of antibodies used.

Target	Source	Vendor	Cat	Lot	RRID	Concentration	Application
GFAP	Mouse	BD pharmaceutical	55328	68139	AB_396366	1/2000	IHC
Fibronectin	Rabbit	Sigma	F3648	032M4752V	AB_476976	1/200	IHC
Mouse IgG	Donkey	Jackson Labs	715066151	124850	AB_476976	1/500	IHC
Rabbit IgG	Donkey	Jackson Labs	711066152	127693	AB_2340788	1/500	IHC
GFAP	Rabbit	Dako	Z0334	20035994	AB_1001338	1/5000	IHC
BrdU	Sheep	Abcam	Ab1893	GR308688-2	AB_302659	1/200	IHC
BrdU	Sheep	Abcam	Ab1893	GR300688-8	AB_302659	1/100	IHC
Sheep IgG	Donkey	Jackson Labs	713066147	117195	AB_234071	1/500	IHC
BrdU	Sheep	Abcam	Ab1893	GR300688-8	AB_302659	1/200	IF
Pu.1	Rabbit	CST	2258	4,5	AB_2186909	1/200	IF
Sox9	Rabbit	Sox9	ab5535		AB_2239761	1/1000	IF
Rabbit IgG	Donkey	Jackson Labs	711586152	146647	AB_2340622	1/500	IF
Sheep IgG	Donkey	Jackson Labs	713546147	139521	AB_2340746	1/500	IF
CD44	Rabbit	Abcam	ab189524	GR320797-10	AB_2885107	1/500	IF
CD44	Rabbit	Abcam	ab189524	GR320797-10	AB_2885107	1/125	ICC
Nestin	Chicken	Novus Biologicals	NB100-1604	NES7877982	AB_2282642	1/7500	ICC
Rabbit IgG	Rabbit	ThermoFisher	A-31572	2017396	AB_162543	1/500	ICC
Chicken IgG	Chicken	Jackson Labs	703-606–155	139628	AB_2340380	1/500	ICC
Nuclear Stain	Hoescht	Invitrogen	2036669	33342		1/500	ICC/IF

Applications: IHC: immunohistochemistry; IF: immunofluorescence; ICC: immunocytochemistry.

### Microscopy and Imaging

Stereology was performed on Zeiss Imager M.2 scopes using Axio MRc or MRm cameras for brightfield or fluorescent imaging, respectively. Information on how each image was acquired can be found in Table 3. Image adjustments (color balance, brightness, contrast) and annotations were performed in Adobe Photoshop (V23).

**Table 3.** Acquisition and processing information for all images shown in paper.

Image(s)	Microscope	Magnification	Numerical aperture	Temperature (C)	Imaging medium	Fluorochromes	Camera	Acquisition software	Processing	Processing software
Figure 1B-C	Zeiss Imager M.2	20×		23	Fluoromount-G (Southern Biotech)	Hoescht, Alexa Fluor 594	AxioCam MRm	Stereoinvestigator (MBF)	“Deep Focus” Deconvolution algorithm	Stereoinvestigator (MBF)
Figure 1D	Keyence bz-x810	60×	1.4	23	Fluoromount-G (Southern Biotech)	Hoescht, Alexa Fluor 594	Keyence bz-x810	Keyence bz-x810 acquisition software	Maximum projection	Adobe Photoshop
Figure 1E	Zeiss Imager M.2	60×	1.4	23	Fluoromount-G (Southern Biotech)	Hoescht, Alexa Fluor 594, Alexa Fluor 647	AxioCam MRm	Axiovision	NA	NA
Fig 2E-F	Keyence bz-x810	60×	1.4	23	Depex (EMS)	NA	Keyence bz-x810	Keyence bz-x810 acquisition software	Stitching of high power images	Keyence bz-x810 analysis software
Fig 3E-F	Keyence bz-x810	60×	1.4	23	Depex (EMS)	NA	Keyence bz-x810	Keyence bz-x810 acquisition software	Stitching of high power images	Keyence bz-x810 analysis software
Fig 4 C-F	Zeiss Imager M.2	60×	1.4	23	Fluoromount-G (Southern Biotech)	Hoescht, Alexa Fluor 594, Alexa Fluor 488	AxioCam MRm	Stereoinvestigator (MBF)	Apotome Deconvolution, Maximum projection	Stereoinvestigator (MBF), Adobe Photoshop

### Isolation, Culture, and Staining of Spinal Cord NSC

Multipotent mouse spinal cord neural stem cells (mNSC) were isolated from 16 day old C57BL/6 spinal cord tissue. Briefly, a 3–5 mm piece of thoracic spinal cord was isolated and dissociated manually into single cells for a free-floating neurosphere culture in Temple DMEM based growth medium supplemented with 10 mg/mL ciprofloxacin. After first passage, mNSC were cultured as monolayers on poly-L-ornithine (PLO)/laminin (LAM) coated vessels in X-vivo 15 (Lonza) based growth medium (GM) supplemented with 20 ng/mL bFGF (Invitrogen PHG0026), 2 ng/mL EGF (Invitrogen PHG0311), 2 µg/mL Heparin (Sigma H-3149), 63 µg/mL (NAC) (Sigma A9165), 10 µg/mL N2 (Invitrogen A13707-01), and 10 ng/mL LIF (Invitrogen PHG0026) as previously described (Benavente et al., 2020; Piltti et al., 2018). mNSC exhibit stable growth rate, sustained neurosphere-initiating capacity, multipotency, and responsiveness to migration cues in vitro similarly to human neural stem cell lines (Uchida et al., 2000). mNSC were fed twice a week. Once at ∼80% confluency, mNSC were passaged to PLO and Laminin coated chamber well slides. After cells returned to ∼80% confluency, media was aspirated from slides, and cells were fixed in freshly thawed 4% PFA for 10 minutes. Cells were washed once with PBS, then blocked in PBS + 5% donkey serum (017-000-121, Jackson Labs), then stained for CD44 (Abcam Cat# ab189524, RRID:AB_2885107) overnight at 4o C. The next day, primary antibody was removed, cells were washed 3 times with PBS for 5 minutes, and secondary antibody was added for 1 hour (slides were subsequently kept in the dark). Cells were again washed 3 times with PBS for 5 minutes, then block in PBS + 5% donkey serum + 0.3% Triton X to permeabilize cell membranes. Cells were then stained for Nestin (Novus Cat# NB100-1604, RRID:AB_2282642) overnight at 4o C. The next day, primary antibody was removed, cells were washed 3 times with PBS for 5 minutes, and secondary antibody and Hoechst (Invitrogen Cat# 2036669) were added for 1 hour. Finally, cells were washed 3 times with PBS for 5 minutes then slides were mounted with Flouromount-G (Southern Biotech). Further details about antibodies used can be found in Table 2.

### Behavior

All tasks were assessed prior to injury, and at 2 and 4 weeks post-injury, and all behavioral analysis was performed by researchers blinded to experimental group. Horizontal ladderbeam performance was assessed as described previously (Anderson et al., 2017). Additional gait analysis was performed using CatWalk XT (Noldus v9.0).

### Statistics and Data Visualization

Statistical analyses and data visualization were performed using R/RStudio, using *the tidyverse, gridExtra, A86, ggpubr, ggsignif, patchwork, raster, tiff, jpeg*, and *png* packages(Auguie, 2017; Constantin & Patil, 2021; Creasman, 2023; Hijmans, 2022; Kassambara, 2020; Müller & Wickham, 2023; Pedersen, 2022; Team, 2020; Urbanek, 2013, 2021; Urbanek & Johnson, 2022; Wickham et al., 2019). Groups and timepoints were compared statistical using a student’s T test or ANOVA with tukey post-hoc test, as described specifically in each figure legend. Ns, standard error, etc. are also reported in each figure legend.

## Results

### CD44 Expression in Naïve WT and KO Spinal Cord

**Figure 1.**
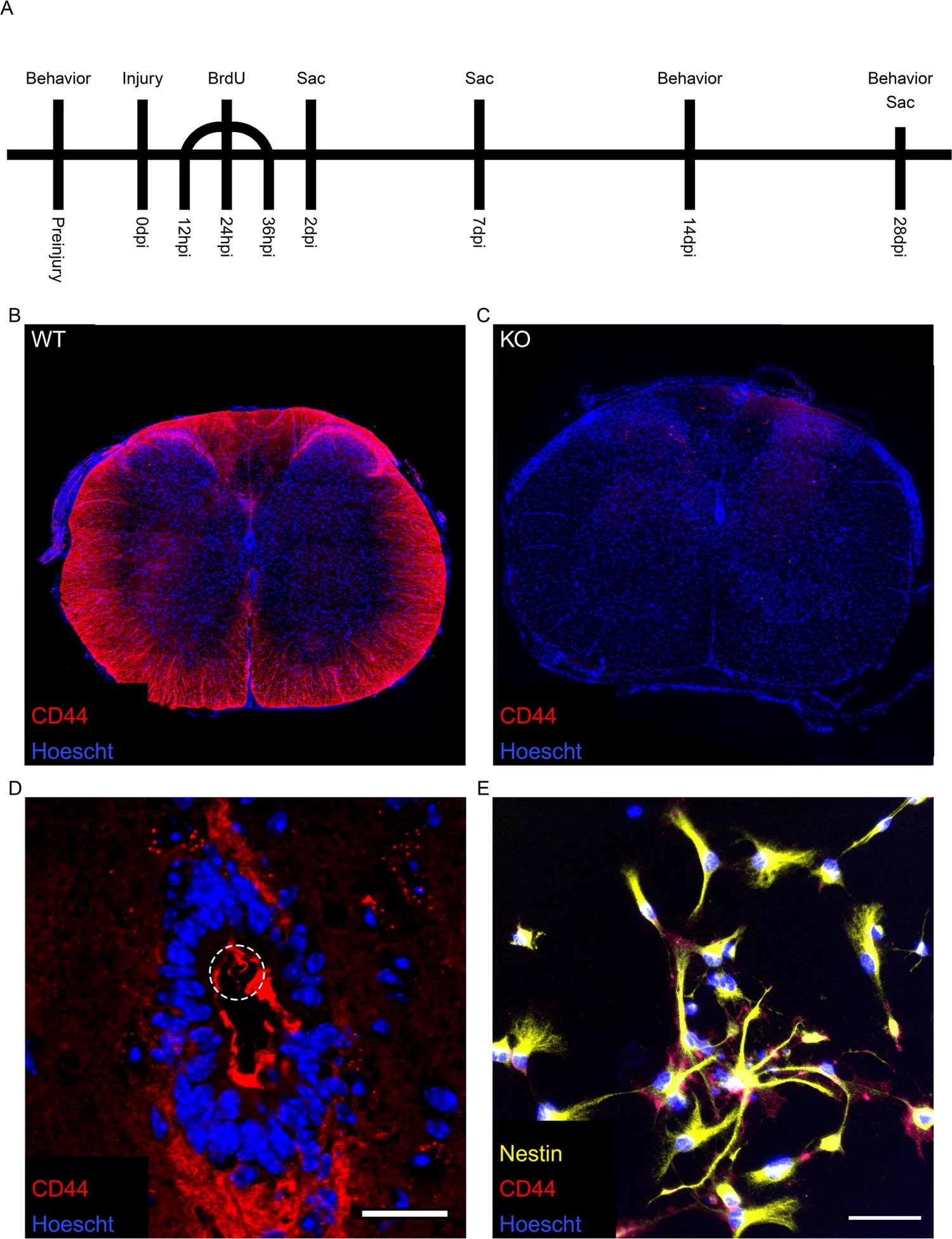
Experimental design and expression of CD44 in the spinal cord. (A) Experimental timeline showing timepoints at which locomotor behavior was collected, BrdU was injected, and animals were sacrificed, perfused, and dissected (Sac). HPI = hours post injury, DPI = days post injury. (B) CD44 expression in the spinal cord. Scale bar = 100 µm. (C) CD44 staining in the spinal cord of CD44 KO mice, showing complete ablation of CD44 expression. Scale bar = 100 µm. (D) High power (60x) image of CD44 expression in the central canal. White circle indicates a CD44-postive process extended from the apical end of a canal-resident NSC. Scale bar = 10 µm. (E) CD44 and Nestin co-labeling in mouse spinal-cord derived NSC. Scale bar = 50 µm.

We first performed immunohistochemistry for CD44 to both confirm the validity of the CD44 KO mice used, and to examine the pattern of CD44 staining in the spinal cord. CD44 labeling was absent in KO mice. Consistent with the published literature demonstrating that astrocytes are a prominent expressor of CD44 in the central nervous system, the pattern of CD44 expression in the spinal cord was most pronounced in the white matter (Figure 1B,C). Labeled cells closely resembled the morphological appearance of astrocytes, and indeed CD44 and GFAP staining strongly colocalize (Supplemental Fig 1). We also identified robust CD44 expression in the ependymal cells of the central canal, which are the endogenous population of NSC in the spinal cord (Figure 1D). Expression of CD44 in spinal cord ependymal cells has not previously been reported, but is interesting in light of the reported contribution of this population to astroglial scar formation after spinal cord stab injuries, combined with identification of C1q-CD44 mediated NSC chemotaxis and high concentrations of C1q present in the blood/serum (Barnabe-Heider et al., 2010; Benavente et al., 2020; Hooshmand et al., 2017; Meletis et al., 2008; Ren et al., 2017; Sabelström et al., 2013; 2014; Stenudd et al., 2015). Accordingly, to validate CD44 expression in spinal cord NSC, we isolated NSC from mouse spinal cord tissue and conducted double-labeling immunocytochemistry of cultured cells for CD44 and the NSC marker nestin, identifying robust colocalization between these markers (Figure 1E). These data confirm the expression of CD44 in both spinal glial and ependymal cells, and the KO status of the mouse model employed.

### CD44 KO Decreases BrdU + Cell Number at the SCI Epicenter

**Figure 2.**
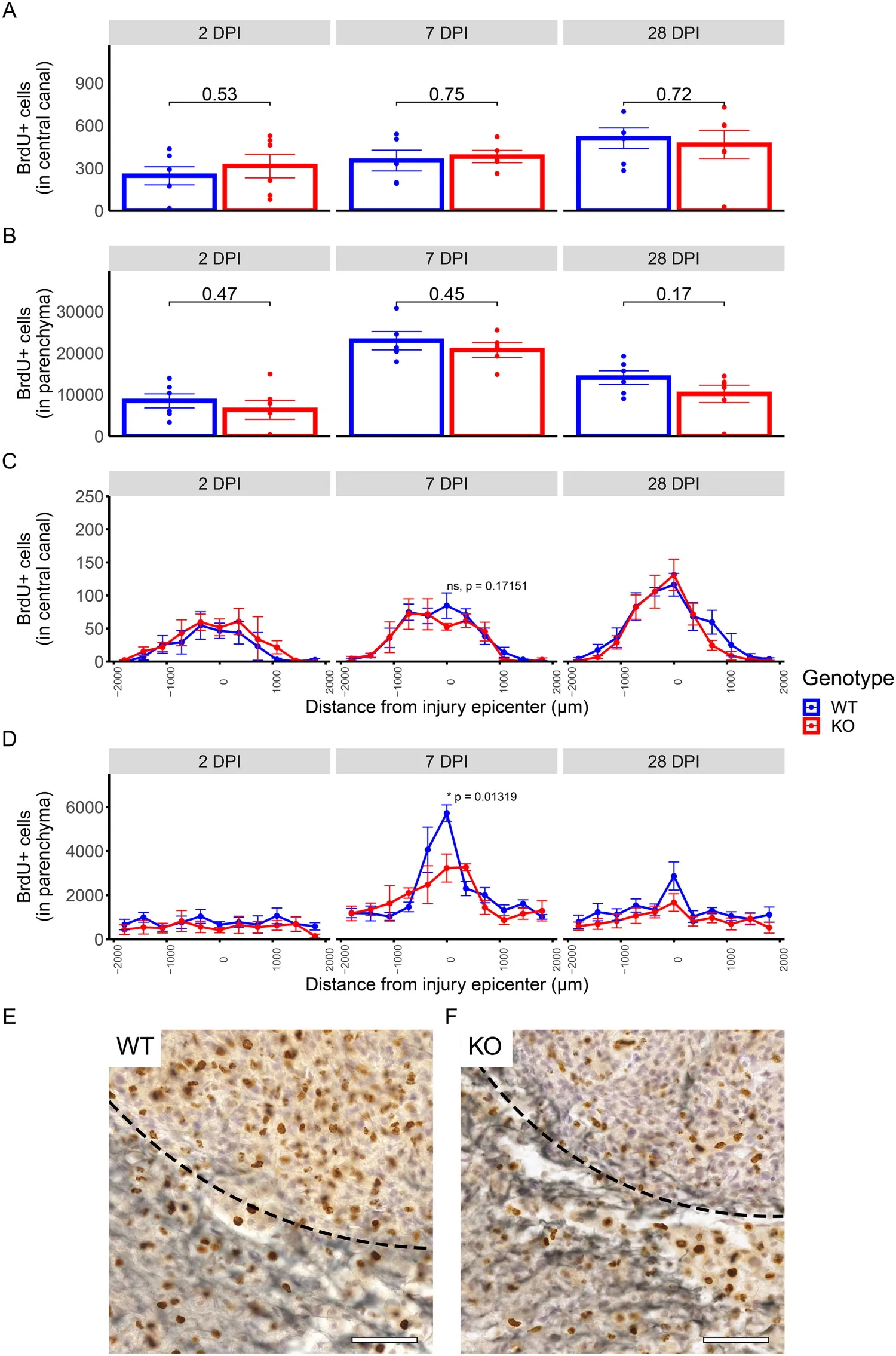
Temporal and spatial distribution of acutely activated, BrdU-labeled cells post-SCI shows distributional differences in WT vs CD44 KO mice at 7 DPI, as determined by unbiased stereology. Time points shown are at 2, 7 and 28 DPI in both WT and CD44 KO mice. (A) Total BrdU + cells in the central canal. (B) Total BrdU + cells in the spinal cord parenchyma. (C) Section by section analysis demonstrating spatial distribution of BrdU + cells in the central canal. (D) Section by section analysis demonstrating spatial distribution of BrdU + cells in the spinal cord parenchyma. (E,F) Images at the injury epicenter of WT and CD44 KO mice, showing BrdU labeling in brown and GFAP labeling in blue. Two-tailed student’s *t-*test as indicated by *P* values listed. Scale bars = 50 µm. Graphs show mean +/− SEM; *N* = 5–6 for each genotype and timepoint.

Previous reports have provided detailed analyses of the proliferation of spinal cord glial and ependymal cells to SCI, and the contribution of these cells to astroglial scar formation, (Barnabe-Heider et al., 2010; Meletis et al., 2008; Ren et al., 2017; Sabelström et al., 2013; 2014; Stenudd et al., 2015). Because the well-established peak of astrocyte proliferation after SCI occurs in the first post-injury week, we focused on whether CD44 KO alters the response of acutely-dividing cells. To investigate this, CD44 WT and KO mice received unilateral C5 cervical contusion injuries followed by BrdU injections at 12, 24, and 36 hours post injury (Figure 1A). Unbiased stereological quantification of the number of cells that exhibited BrdU incorporation was conducted at 2, 7 and 28 dpi, with analysis of total BrdU + cells adjacent to the C3-7 central canal and in the spinal parenchyma. BrdU labeling in both CD44 WT and KO mice was detectable at all timepoints examined (Figure 2). Two-way anovas for genotype and time (followed by Tukey post-hoc test) revealed that genotype did not significantly affect total BrdU + cell number in either the parenchyma or the central canal region. The same analyses also revealed that in the canal region, overall BrdU + cell numbers are significantly increased from 2 to 28 dpi (*p* = 0.0250391), whereas in the parenchyma they significantly increase from 2 to 7 dpi (*p* = 0.0000002) and then significantly decrease from 7 to 28 dpi (*p* = 0.0001245).

While total BrdU + cell number in these regions did not differ by genotype, the spatial distribution of BrdU labeling was markedly different in the spinal parenchyma at 7 dpi, with CD44 KO mice exhibiting reduced BrdU labeling specifically at the injury epicenter (Figure 2D). The spatially focused difference in BrdU + cell localization, established function of CD44 in cell migration, and abundance of chemotactic cues (e.g. C1q) at the injury epicenter (Benavente et al., 2020; Hooshmand et al., 2017), suggest that CD44 KO impairs the migration of proliferating cells towards the injury site. Alternatively, CD44 KO could impair the proliferative response of a cell population localized proximal to the injury site.

CD44 KO does not alter the number of BrdU + cells co-labeled for GFAP, but does alter scar formation

We next investigated how reduced BrdU + cell number at the SCI epicenter in CD44 KO mice affected astroglial scar formation. Scar formation is a critical part of the post-SCI response, requiring both recruitment and alignment of newly generated astrocytes. Failures in this process result in significantly worse SCI outcomes (Anderson et al., 2016; O’Shea et al., 2017; Sabelström et al., 2013; Stenudd et al., 2015; Wanner et al., 2013). To determine if recruitment of acutely-dividing astrocytes was altered in CD44 KO mice, we conducted double immunohistochemical labeling for GFAP+ and BrdU+. No GFAP+/BrdU + cells were detected in either genotype at 2, 7, or 28 dpi in this unilateral contusion model, suggesting that whatever astrocyte proliferation occurred happened outside the 12–36 hour post-injury time window. There is evidence to suggest that the bulk of astrocyte proliferation occurs within the 2–7 time period, which would explain our inability to detect GFAP+/BrdU + cells (Amat et al., 1996; Bush et al., 1999; Faulkner et al., 2004; Norton et al., 1992; Zai & Wrathall, 2005).

**Figure 3.**
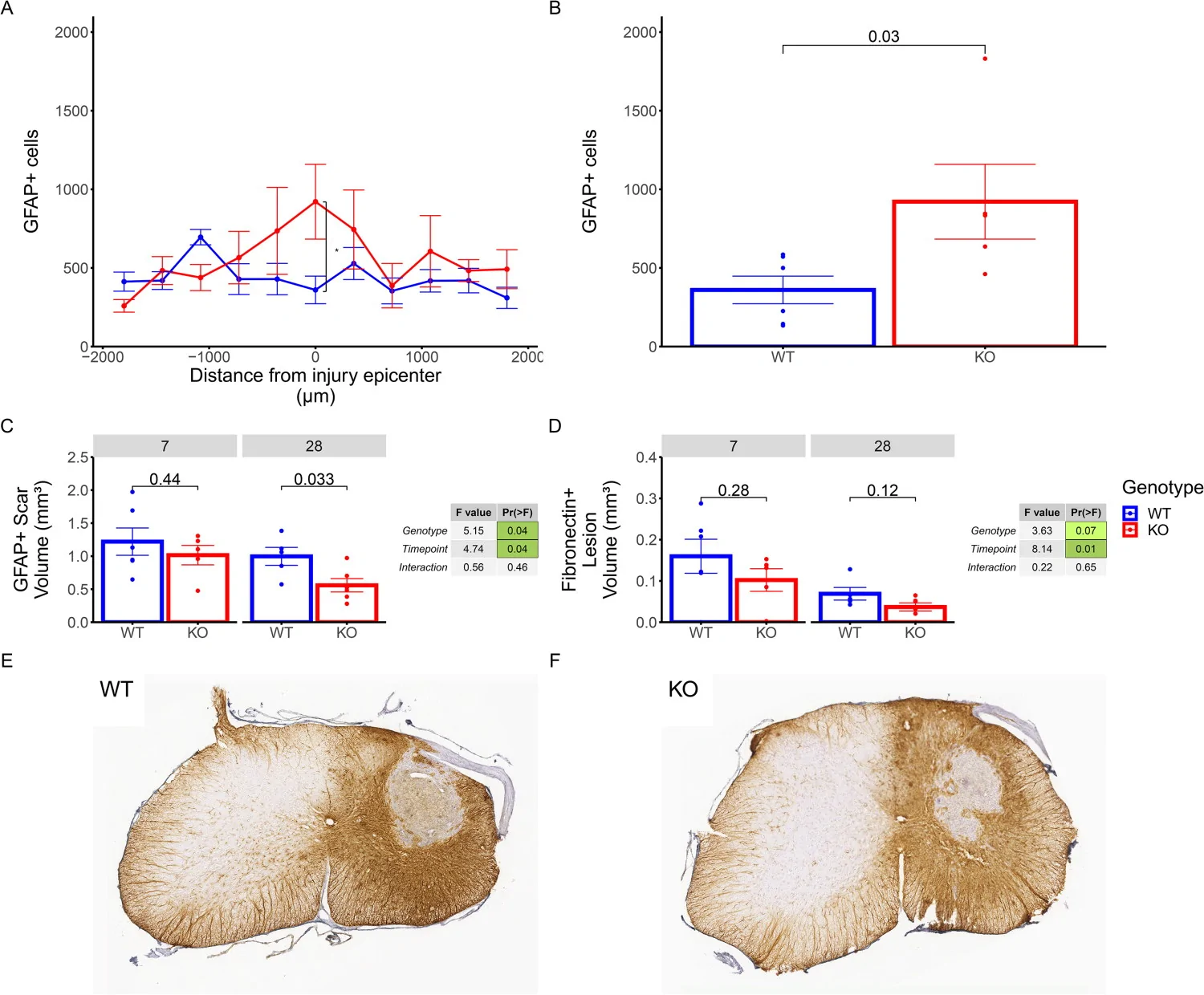
Scar formation post-SCI is altered in WT vs. CD44 KO mice, as determined by unbiased stereology. (A) Section by section analysis demonstrating spatial distribution of GFAP+ cells at 7DPI. (B) Total GFAP+ cells in epicenter sections at 7 DPI. (C) GFAP+ volume at 7 and 28 DPI. (D) Fibronectin + volume at 7 and 28 DPI. Two-way ANOVA tables for time and genotype. (E,F) Images at the injury epicenter of WT and CD44 KO mice at 28 dpi, showing GFAP labeling in brown. Scale bar = 100 µm. Two-tailed student’s t-test as indicated by P values listed. Graphs show mean +/− SEM. *N* = 5–6 for each genotype and timepoint.

These data suggest that the BrdU + population of proliferating cells at the lesion epicenter does not yield GFAP+ cells, however, astroglial scar formation could still be altered by a change in migration/recruitment in CD44 KO mice. We tested this hypothesis by determining the number and distribution of all astrocytes, with or without BrdU labeling. While the total numbers of GFAP+ astrocytes was not different across genotypes, the spatial distribution was again significantly different between genotypes at 7 dpi. In contrast to our BrdU data, however, CD44 KO mice exhibited a sharp peak of astrocytes at the injury epicenter in comparison with WT mice (Figure 3A,B). Based on this observation we next analyzed scar morphology and lesion size by unbiased stereology using the Cavalieri probe to determine GFAP+ and fibronectin + volume. Surprisingly, while lesion volume remained unchanged between genotypes, scar volume was decreased in CD44 KO mice, despite the increased number of astrocytes at the lesion epicenter (Figure 3C,D). Together, these data suggest that CD44 KO leads to an increase in the recruitment of GFAP+ astrocytes to the lesion site, accelerating compaction of the astroglial scar, and identifying a complex modulation of astrocyte behavior.

**Figure 4.**
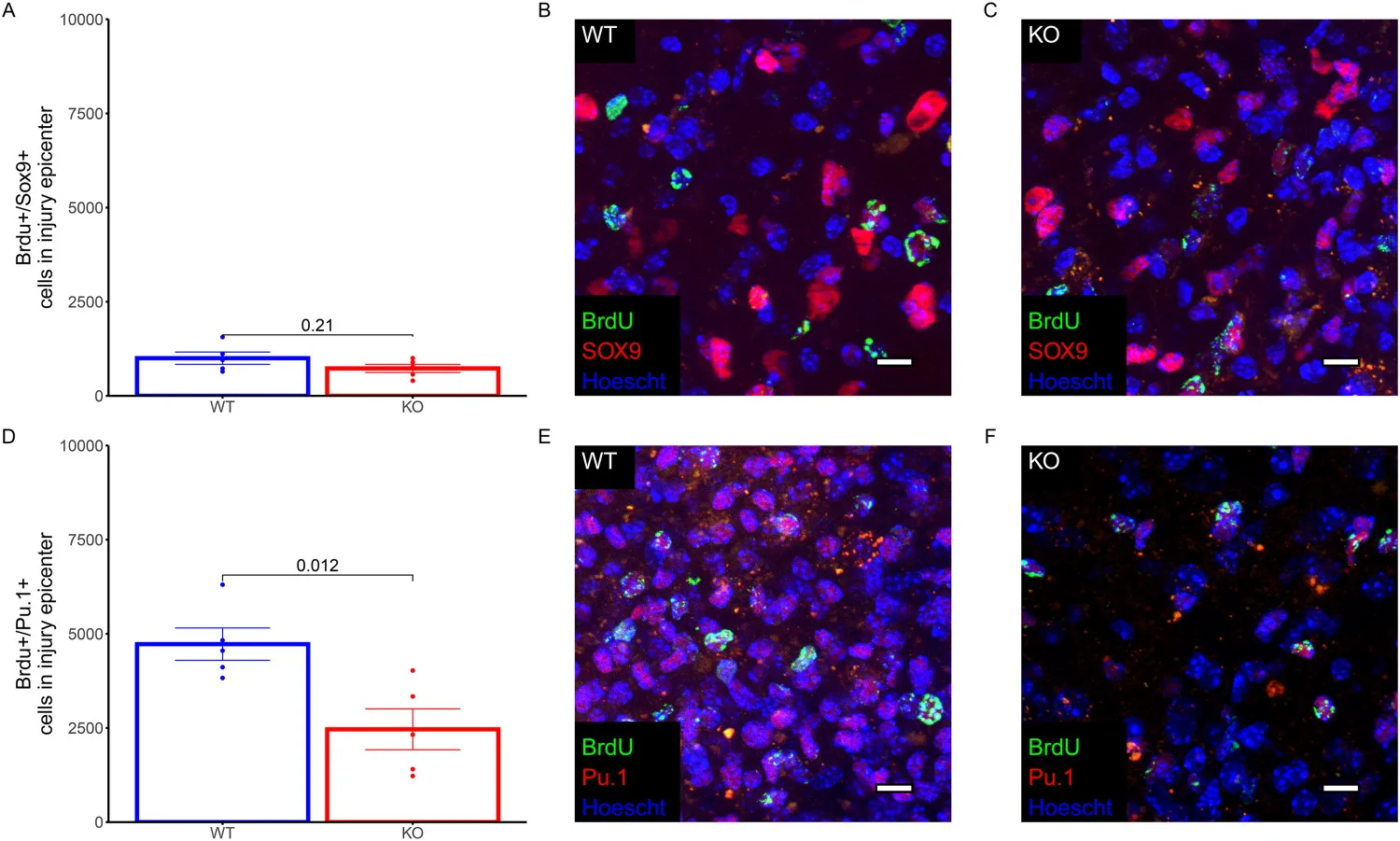
BrdU-labeled cells at the SCI epicenter at 7 DPI are primarily Pu.1+ and these Pu.1+/BrdU + cells are reduced by CD44 KO. (A) Total SOX9+/BrdU + cells in the injury epicenter. (B,C) Images at the injury epicenter of WT and CD44 KO mice, with arrows indicating examples of BrdU and SOX9 colabeling. (D) Total Pu.1+/BrdU + cells in the injury epicenter. (E,F) Images at the injury epicenter of WT and CD44 KO mice, with arrows indicating examples of BrdU and Pu.1 colabeling. Two-tailed student’s *t*-test as indicated by *P* values listed. Graphs show mean +/− SEM. *N* = 5–6 for each genotype. All scale bars = 10 µm.

Fewer acutely activated microglia/macrophages are present in the SCI epicenter at 7DPI in CD44 KO mice

Under normal post-lesion conditions, the astroglial scar condenses over time, creating a barrier between the lesion core and spared tissue, a process orchestrated by microglia {Bellver-Landete, 2019 #36}. Accordingly, we next sought to determine the identity of the BrdU labeled cells at the 7 dpi injury epicenter. Based on the known role of microglia in scar orchestration and our observation of CD44 expression in the ependymal stem/progenitor populations. We used Pu.1 as a marker for microglia and myeloid-derived cells and SOX9 as a marker for astrocyte progenitors (Sun et al., 2017; Walton et al., 2000). We found that these two markers accounted for most BrdU-labeled cells in the injury epicenter of both genotypes; approximately 20% of BrdU-labeled cells in the injury epicenter section were SOX9 positive, and 80% were Pu.1 positive. KO animals exhibited a significant reduction in BrdU+/Pu.1+ Cells (*p* = 0.012), but no significant change in total BrdU+/SOX9+ cells (*p* = .21), vs. WT littermates. These data indicate that myeloid cells were both the primary population labeled by BrdU under this labeling/injury paradigm, and the primary cell group for which acute recruitment to the epicenter site was affected by CD44 ablation. This aligns with previous data on the time course of myeloid cell (specifically microglial/macrophage) infiltration post-SCI, and with the known role CD44 has in immune cell migration/infiltration (Beck et al., 2010; Cichy & Puré, 2003; Ponta et al., 2003).

## CD44 KO Results in Beneficial Locomotor Outcomes

**Figure 5.**
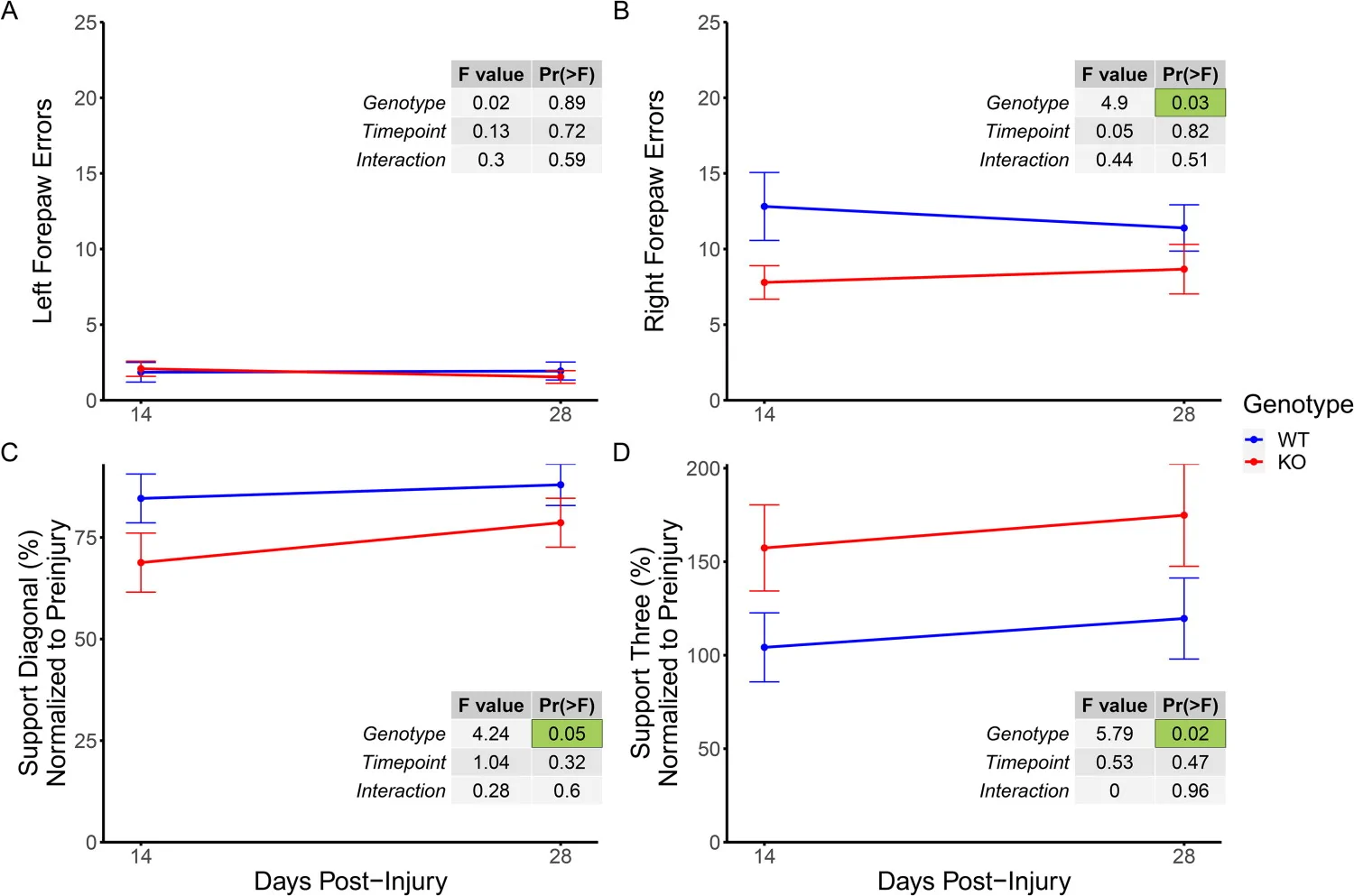
Functional locomotor recovery post-SCI demonstrates improvement in CD44 KO vs. WT mice. Horizontal ladder beam errors for the Left (A) and right (B) forepaw. CatWalk kinematic analysis of percent of time spent on diagonally positioned paws (C) or three paws (D). Two-way ANOVA tables for time (repeated measures at 14 and 28 DPI) and genotype shown for each statistic (*n* = 8–10 per genotype).

To determine the effect that these changes in cellular distribution had on locomotor outcome, we evaluated performance on a horizontal ladderbeam task at 14 and 28 dpi. CD44 KO mice exhibited a decrease in the number of errors vs. WT littermates, suggesting a net locomotor benefit of CD44 KO (Figure 5B). Catwalk kinematic gait analysis supported this result, identifying that CD44 KO mice spent more of their time walking in a “diagonal” arrangement of their paws vs. having three paws on the ground at once (Figure 5B-C). The “diagonal” paw arrangement is more common pre-injury, and is indicative of a normal gait where animals are able to balance properly, whereas animals that are injured will need to have three paws on the ground a greater percentage of the time in order to compensate for their lack of function and balance. Thus, the CD44 KO mice’s comparatively higher rate of “diagonal” paw placement vs WT littermates is further evidence of CD44 KO resulting in benefits to locomotor outcomes.

## Discussion

Given the debilitating nature of SCI and the healthcare costs associated with this type of traumatic injury, there is a need for a better understanding of the fundamental cellular mechanisms in play during the post-SCI response. CD44 is a critical regulator of cellular behavior, and a potential therapeutic target for a post-SCI intervention. Here, we provide evidence that ablation of CD44 results in marked changes to the post-SCI response, altering the behavior of astrocytes and immune cells, and that the net result of these changes is protective in terms of animal locomotor ability.

CD44 has long been established as regulator of cellular migration and cell-cell matrix interactions in a variety of cell types and tissues (including in the CNS), and our results align with that established literature (Chetty et al., 2012; Cichy & Puré, 2003; Dzwonek & Wilczynski, 2015; Konopka et al., 2016; Morath et al., 2016; Yan et al., 2015). Additionally, it has been shown that CD44 also modulates NSC migration both in vitro and after transplantation into the injured spinal cord, highlighting the importance of this molecule in the cellular response to neurotrauma (Benavente et al., 2020). Here we investigated how CD44 modulates the endogenous cellular response to SCI. The microenvironment and extracellular matrix are drastically different post-SCI vs in a homeostatic cord, posing numerous questions for how CD44 will respond to these changes and how this will effect CD44-expressing cells (Benavente et al., 2020; Dolma & Kumar, 2021; Dzwonek & Wilczynski, 2015; Fan et al., 2018; Figley et al., 2014; Galvan et al., 2008; Peterson & Anderson, 2014).

Given that CD44 plays so many roles and is expressed so broadly, it makes sense that our results are complex and multi-faceted. We see a clear difference in recruitment of acutely-activated cells to the injury epicenter. Interestingly, though, we determined that while ∼20% of acutely-activated cells were Sox-9 positive stem/progenitor cells, typically restricted to the astrocyte lineage, these cells were not the population for which recruitment to the epicenter was affected. Instead, Pu.1+ immune cells were the much more significantly affected population, with significantly fewer reaching the epicenter in CD44 KO mice vs. WT. Importantly, while both cell types have to migrate after injury, many immune cells present post-SCI are not native to the cord, and thus have to infiltrate into the cord via the periphery. This longer journey to the cord could make these cells more reliant on CD44 to reach their target, and thus more likely to be affected by its absence (Beck et al., 2010; Johnson & Ruffell, 2009; Smadja-Joffe et al., 1996).

Adding to this complexity is the fact that at none of the timepoints surveyed did we see significant numbers of GFAP+/BrdU + cells, meaning that not only were astrocytes not proliferating at the 12–36 hour window in which we injected BrdU, but that the Sox9+/BrdU + cells we did label did not differentiate into mature astrocytes. This occurred despite robust BrdU-labeling of central canal cells, which have been proposed to contribute to the glial scar post-SCI (Barnabé-Heider et al., 2010; Meletis et al., 2008; Sabelström et al., 2013, 2014; Stenudd et al., 2015). Given the distribution of Sox9+/BrdU + cells around the perimeter of the lesion, it is possible that these cells contributed to scar formation in a way that did not involve differentiation into mature astrocytes. It is also critically important to acknowledge the role of injury model and labeling paradigm, as studies that vary these parameters have reported contrasting results regarding the contribution of different cell populations to glial scarring (Barnabe-Heider et al., 2010; Li et al., 2018; Meletis et al., 2008; Ren et al., 2017; Sabelström et al., 2013, 2014). In this regard, prior studies have primarily utilized either crush or hemisection injuries, in contrast to the contusion lesion employed here.

While we did not observe BrdU labeling of mature astrocytes, we did still detect significant changes to the astrocytic response to SCI. This included a significant increase in astrocyte numbers at the injury epicenter at 7DPI in CD44 KO vs. WT mice, and a significant decrease in the long-term GFAP+ volume in CD44 KO mice. These results might initially seem contradictory, as one would expect an increase in astrocyte number to correspond to an increase in GFAP+ volume. However, GFAP+ volume is highly sensitive to factors like astrocyte hypertrophy and other morphological changes astrocytes undergo post-SCI. Scar formation is a multi-cellular, multi-phase process (Bellver-Landete et al., 2019; Wanner et al., 2013; Zhang et al., 2021). This, coupled with the fact that CD44 KO was global, is consistent with a complex impact of CD44 on scar formation, as CD44 KO impacted not only astrocytes, but also the cells that astrocytes interact with and resulting molecular environment. These data therefore identify a need to dissect the role of CD44 in astrocyte behavior post-SCI, which will require a combination of cell-specific KO and pharmacological inhibitor approaches, along with more robust techniques for tracking scar formation (Wanner et al., 2013). Regardless, however, our behavioral results indicated CD44 KO improved functional locomotor recovery, demonstrating that the aggregate effect of global CD44 KO was a positive one.

The effect of CD44 on immune cell behavior was particularly interesting. Recruitment of Pu.1+/BrdU + cells was dramatically at 7DPI was dramatically altered, at timepoint that marks a critical influx/increase of macrophages and microglial in the injured cord (Beck et al., 2010). Given both the established role of CD44 in altering immune cell behavior, and the critical importance of the immune response in altering SCI outcomes, we hypothesize that immunomodulatory changes due to CD44 KO drive the protective effects observed. As noted above, addressing this question will require a more cellularly and temporally specific CD44 KO model. Critically, previous studies have shown that decreasing microglia recruitment post-SCI specifically leads to a disorganized scar, exacerbated injury, and worsened locomotor outcomes (Bellver-Landete et al., 2019; Fu et al., 2020; Zhou et al., 2023). This would seem in conflict with our data showing decreased recruitment of Pu.1+/BrdU in CD44 KO mice correlates with improved locomotor outcomes and more resolved scarring. However, the Pu.1+ population is a mix of macrophages and microglia, suggesting that microglia and infiltrating macrophages/monocytes could exert differential roles in astroglial scar formation and organization. In this regard, previous studies have suggested that macrophage depletion exerts a beneficial effect on locomotor recovery (Ding et al., 2021; Milich et al., 2019; Popovich et al., 1999). Beyond simply affecting cellular recruitment, there is also the potential for CD44 to have deeper mechanistic effects, altering the factors cells secrete or otherwise shifting cellular fate and/or function. Another, additional avenue of research not explored here is the potential for CD44 KO to result in neuroprotective effects, either directly via altering neuronal cells, or indirectly through influencing other cells to secrete neuroprotective factors. Due to this complex nature, future studies will be needed to fully disentangle the role of CD44 on each unique cell population and explore these potential mechanisms in greater depth. Indeed, a recent study has highlighted the use of a CD44 function-blocking antibody as a means of altering post-SCI scarring and improving locomotor outcomes (Guo et al., 2024). This, combined with our data, highlights a potential elucidating the mechanisms at play and further develop the transitionally potential of these approaches.
